# Renal Dysfunction due to Tenofovir-Diphosphate Inhibition of Mitochondrial Complex V (ATP Synthase)

**DOI:** 10.1093/function/zqad010

**Published:** 2023-02-24

**Authors:** Nicolas Sluis-Cremer

**Affiliations:** Department of Medicine, Division of Infectious Diseases, University of Pittsburgh School of Medicine, Pittsburgh, PA 15213, USA

## A Perspective on “Integration of High-Throughput Imaging and Multi-Parametric Metabolic Profiling Reveals a Mitochondrial Mechanism of Tenofovir Toxicity”

Prescription drugs are a common cause of kidney injury. Drug-induced nephrotoxicity, however, is a complex process, and likely involves a combination of factors, including (i) drug characteristics (eg, solubility, structure, and charge); (ii) drug dose and duration of therapy; (iii) inherent drug toxicity; (iv) renal metabolism and excretion of the drug; and (v) patient characteristics that enhance their risk for kidney injury. The mechanisms of drug-induced nephrotoxicity and prevention strategies have been reviewed extensively elsewhere.^1,2^ Tenofovir disoproxil fumarate (TDF) is a nucleoside reverse transcriptase inhibitor used to treat HIV and HBV infections. TDF therapy, however, has been associated with renal impairment, characterized by a decline in glomerular filtration rate and proximal tubular dysfunction.^3^ TDF is a prodrug that is rapidly metabolized to the active component tenofovir in plasma. In cells, tenofvoir is metabolized to its active diphosphate form by adenylate monophosphate kinase (tenofovir monophosphate) and 5′-nucleoside diphosphate (tenofovir diphosphate).^4^ Renal injury is likely related to intracellular tenofovir accumulation in proximal tubule cells. A molecular mechanism of TDF-induced renal toxicity, however, is lacking, but it is thought to be via mitochondrial depletion and structural change, including size and shape changes, and leakage of mitochondrial proteins into the cytosol, with resultant DNA damage, which may even induce apoptosis of the cell.

In a recent study, Pearson *et al*. developed an innovative approach to screen for disease-related functional defects in RPTEC/TERT1 cells, a well-differentiated human-derived cell line that replicates many of the major characteristics of proximal tubular kidney cells in *vivo*.^5^ The RPTEC/TERT1 cells were exposed to TDF, and high-throughput imaging was used to generate quantitative readouts of solute transport and mitochondrial morphology, which facilitated development of treatment protocols that reproduced well-described features in patients. By using multiparametric metabolic profiling, including metabolomic screening, oxygen consumption measurements, and RNA-sequencing, the authors determined a molecular fingerprint of TDF toxicity. They found that TDF results in a dose-dependent decrease in mitochondrial ATP synthase, or complex V (EC 3.6.3.14) activity and expression, whereas other mitochondrial functions and pathways were well preserved. Tenofovir disphosphate was found to directly inhibit complex V. Downregulation of complex V expression was also observed in human biopsies. Complex V synthesizes ATP from ADP in the mitochondrial matrix using the energy provided by the proton electrochemical gradient, and mutations in complex V give rise to severe mitochondrial disease phenotypes, ranging from neuropathy, ataxia, and retinitis pigmentosa to maternally inherited Leigh syndrome.^6^ Of note, in a rat model of TDF nephrotoxicity, the activities of the electron chain complexes I, II, IV, and V were also found to be inhibited.^7^ Collectively, these studies suggest that modulation of complex V activity and expression by TDF is a plausible mechanism for nephrotoxicity.

There are, however, limitations associated with the Pearson *et al*. study. After oral absorption, TDF is rapidly converted to tenofovir in the plasma, and then intracellularly to the active tenofovir diphosphate. Tenofovir, and not TDF, is the primary metabolite that is excreted renally. In this study, the authors used very high concentrations of TDF (300–500 µm). The negative charges of the phosphonate moiety of tenofovir reduce its cellular permeability, while TDF was developed to improve tenofovir permeability. Indeed, in antiviral assays in MT-2 cells, the inhibitory activity of TDF toward HIV-1 is 100-fold greater than tenofovir, but its toxicity is also 120-fold higher.^8^ Tenofovir alafenamide (TAF) is a next-generation tenofovir prodrug that has also been approved for treatment of HIV infection. TAF has greater intestinal and plasma stability than TDF and is associated with a mean 91% lower plasma tenofovir exposure.^9^ Unlike tenofovir, TAF is minimally eliminated in urine, and several clinical trials have shown substantially reduced nephrotoxicity associated with TAF therapy.^10^ Compared to tenofovir, TAF also penetrates cells more efficiently, and one suspects that if this drug was used in the experimental setup described by Pearson *et al*., it would likely also demonstrate nephrotoxicity. Thus, the authors missed an important opportunity to directly evaluate tenofovir activity, and elucidate whether inhibition of complex V occurred at clinically relevant concentrations of this metabolite.

Nevertheless, from a technological perspective, Pearson *et al*. developed an innovative approach that could be used routinely to investigate the mechanisms of nephrotoxicity of others drugs, particularly those in development, and could also show utility for uncovering novel genetic regulators of transport and metabolism in epithelia.
